# MRI for premature neonatal brain injury: a case report

**DOI:** 10.1002/jmrs.226

**Published:** 2017-03-06

**Authors:** Alexander Langham

**Affiliations:** ^1^ Canterbury District Health BoardChristchurchNew Zealand

**Keywords:** Brain injury, MRI, paediatric, ultrasound, white matter

## Abstract

This case report aims to extend analytical thinking and clinical reasoning of clinicians and radiographers when presented with diagnosing premature neonatal brain injuries (PNBI). The report considers the uses and merit of magnetic resonance imaging (MRI) in the primary assessment of PNBI. The traditional technique of cranial ultrasound as the first modality of choice can have several limitations, which includes a lower temporal resolution in its ability to differentiate grey‐white matter distribution patterns, lower spatial resolution in its ability to accurately map white matter fibre tracts and distribution patterns which are critical in white matter injury pathological events. In this specific case report, MRI was useful for the assessment of haemorrhagic brain injury post partum.Therefore, should MRI be considered, the primary imaging modality in these cases when the concerns about PNBI is presented? This case study explores the current trends in MRI neonatal brain imaging and advancements being made in this field.

## Introduction

Magnetic resonance imaging (MRI) for premature neonatal brain injuries (PNBI) is a recurrent request that we face at many clinical centres in Australia and New Zealand in my experience. Our team of neonatologists, paediatricians and paediatric radiologists utilise radiology and its modalities for correct and timely diagnosis of PNBI. Commonly a routine cranial ultrasound is performed, however, ultrasound has limited temporal and spatial resolution when attempting to image white matter injuries. Cranial MRI of preterm neonates is often a more beneficial imaging modality.^1^ This high‐risk group of neonatal premature patients often has associated low birthweight which later in life may be associated with developmental coordination disorders, which according to a recent study was found to affect 40% of low birthweight neonatal preterm children at school age.^2^ As well cognitive impairment and/or language delays have been reported in 20–40% of school‐age children born with very low birthweight.^3^, ^4^, ^5^, ^6^, ^7^, ^8^, ^9^, ^10^, ^11^ This specific case report and discussion is based on a premature low birthweight brain injury patient who had a cranial ultrasound examination performed, when he was post‐partum 32 weeks, and subsequently a feed and sleep MRI scan was performed to determine the pathological diagnosis of PNBI. The use and access of MRI for this type of injury is commonplace today, with most systems diffusion tensor imaging (DTI) capable for distributional white matter tract imaging.

## Consent

The patient's caregiver has provided consent for his case to be reported and published.

## Case Report

A patient presented from the Neonatal Intensive Care Unit for MRI following concerns that they had experienced PNBI as a result of traumatic brain haemorrhage, following delivery. A previous cranial ultrasound study was inconclusive other than some enlarged hydrocephalus. MRI was utilised to investigate this neonate more intensively.

A routine paediatric neonate head MRI protocol was performed on a General Electric (GE) Signa HDxt 1.5 Tesla (GE MRI) System using an 8 channel GE head coil. A vacuum beanbag was used to support and minimise head motion. The child was fed and a sucrose solution given prior to the scan to calm them down for sleep, which is described as a feed and sleep type scan. A routine neonatal paediatric head protocol was used, which includes the following sequences;

Localiser 3‐plane scout

T2 transverse (TR3400, TE104)

T2* transverse (TR 345, TE20, FA30)

DWI b0‐b1000 transverse (TR 9200, TE89)

T1 sagittal (TR 430, TE11)

T2 coronal (TR 4200, TE 104)

## Radiologist Report

Post‐partum ultrasound report: “no obvious appearance of germinal matrix periventricular haemorrhage”. The lateral, third and fourth ventricles are larger than normal ranges which suggest, there is something pathological occurring in this specific case. There was therefore a need for a cranial MRI to evaluate the presence of PNBI.

MRI report: “Marked ventricular dilation of the lateral, third, fourth ventricles, extending into a prominent infra cerebrospinal fluid space. This is likely to be secondary to extensive intra‐ventricular haemorrhage which arises from germinal matrix/choroid plexus regions. Cerebellar atrophy, but no definite structural abnormality of the cerebellum is seen.” This MRI report demonstrates that haemorrhage due to PNBI is visualised in the lateral, third and fourth ventricles, structurally the brain ventricles are enlarged. Pathologically it demonstrates PNBI with specific periventricular leukomalacia (PVL) injury.

## Discussion

**Figure 1:**
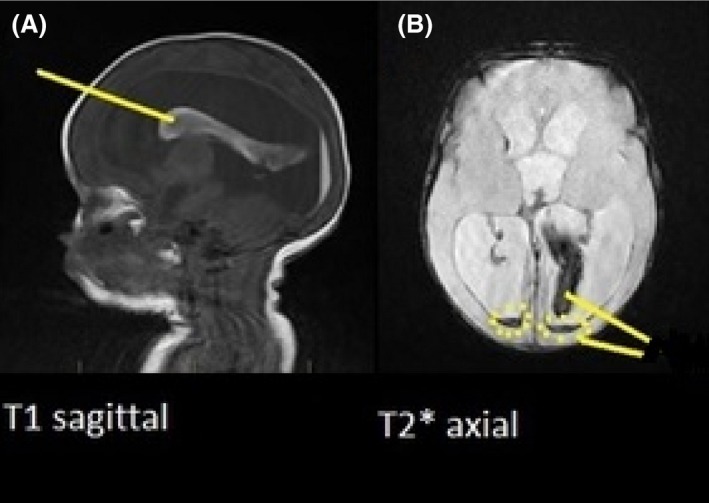
(A) T1‐weighted saggital, hyperintensity showing haemorrhagic product. (B) T2 gradient transverse, hypointesnity showing ferritin heamorrhagic products.

MRI demonstrated PNBI accurately in this case report, haemorrhage is clearly visualised on the examination images, in comparison to the previous ultrasound study performed. It was also able to diagnoses PVL injury more accurately. PNBI consists of multiple mechanisms these are mainly, periventricular haemorrhagic infarction and PVL.^12^ Haemorrhagic injury is demonstrated in this case with high/low signal intensities seen on the MRI images, T1 sagittal and the T2* gradient transverse, (Fig. 1A and B). This hypo‐intensity demonstrates haemorrhagic layering in the occipital horns of the lateral ventricles.

**Figure 2:**
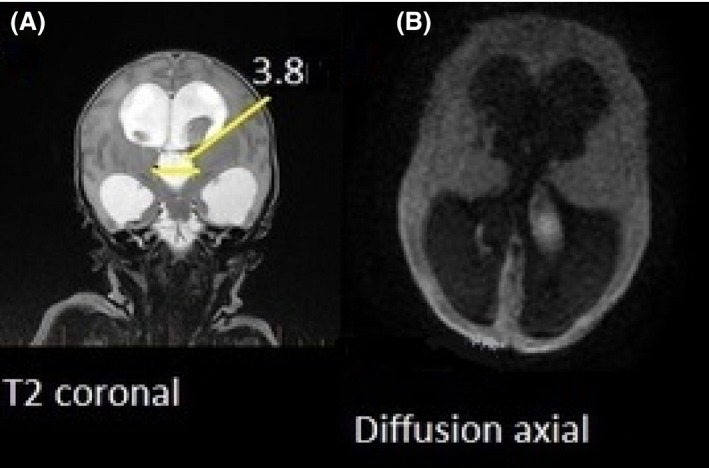
(A) T2 coronal, demonstrating expansive hydrocephalus. (B) Diffusion‐weighted b1000 transverse, limited by artefact minimal air–water–tissue interface.

The T2 coronal (Fig. 2A) MRI image demonstrates the large expanded choroid plexus bilaterally and extensive hydrocephalus.

The diffusion‐weighted transverse MRI image with a bipolar gradient value of b1000 (Fig. 2B) was difficult to interpret in this case as such thin cerebral mantle is present, due to the expansive hydrocephalus, fluid–air–tissue interface artefact and real restricted diffuse areas are so proximal to the skull assessment of diffusion restriction is limited.

In this case, germinal matrix intra‐ventricular haemorrhage has occurred, and secondary to that there is post‐haemorrhagic hydrocephalus which may likely additionally be associated with PVL white matter injury. This imaging highlights the importance of the use of MRI in diagnosing PNBI. Recent literature in peri/prenatal risk factor patterns of PNBI concluded that measured prenatal risk factors did not predict the pattern of brain injury. Additionally, they could not predict the clinical and 30 months neuro‐developmental outcomes or patterns of brain injuries in neonatal infants.^13^ Current research into PNBI, using histopathological biomarkers are now being utilised to predict outcomes and this is a developing field of neonatal medicine.^14^, ^15^ New advancements in MRI DTI techniques at higher field strengths of 3 Tesla and above, enable white matter tract imaging of higher quality. These are utilised to see the distribution of white matter tract patterns, which have significant associated long‐term outcomes for neonatal brain injury patients. Severe white matter injury such as PVL usually results in spastic diplegic cerebral palsy.^16^ Other areas of development in this specific field include histological biochemical mapping, which is looking at the inflammatory response to injury of white matter. This is advancing technology, and so far few have shown promising results.^14^, ^15^ In this specific case report, the neonatal team was primarily looking for structural pathological injury and haemorrhage which was demonstrated well with MRI.

Ultrasound is useful in some abnormal structural injury cases as it has the benefits of being widely available, accessible and cost‐effective in comparison to MRI. Being able to perform multiple cerebral ultrasounds over the duration of the patients stay by definition would increase its sensitivity and specificity on first look. However, literature suggests increased frequency of performing cranial ultrasound was not found to increase the detection of white matter injuries.^1^ Diagnosis of white matter injuries by ultrasound is troublesome and correlation of peri‐ventricular echo densities often correlates with white matter injuries, however, a lack of peri‐ventricular echo densities does not rule out the absence of injury.^1^ Cranial ultrasound is very useful in determining long‐term survival rates of PNBI patients, even if it is unable to detect white matter injuries accurately. This demonstrates that this is not a very useful screening tool for referral for an MRI examination, hence MRI should be considered for all PNBI cases.

With the advancements in critical neonatal care medicine, survival of preterm infants is associated with increased rate of neurodevelopmental impairment, which is currently 3–4 times that of the general population.^17^ The incidence of cerebral palsy in preterm infants is estimated to be 10–15%.^13^, ^18^, ^19^

The mortality rate for childhood stroke is about 1 per 4000 and rates are highest in infants under the 1‐year‐old age group.^17^ More than 95 percent of infants who have a neonatal stroke survive to adulthood, and many have a residual motor or cognitive disabilities. Early diagnosis of PNBI is important as rational intervention plans may prevent or reduce the incidence of lifelong disabilities such as cerebral palsy, epilepsy, and behavioural and learning disorders. In this specific case report, referral to the paediatric neurosurgical team where a surgical shunt intervention was performed to relieve hydrocephalus and the resultant intracranial pressure. Follow‐up MRI will be needed to evaluate white matter tract development at term, 3, 6 and 12‐month follow up MRI examinations with specific white matter tract imaging sequences.

A recent MRI study, using DTI focused specifically on white matter tract migratory development and found that perinatal cerebral white matter injury seems to have major deleterious effects on the subsequent development of fibre tracts both in the cerebral white matter and more cortically. The ultimate impact of PNBI in the new‐born should be considered as a function not only of tissue destruction but also of impaired subsequent brain development.^19^, ^20^ In essence, the long‐term developmental effects of neonatal brain injury are widely unknown. Furthermore, brain MRI scans are commonly performed at a near‐term age in very preterm infants and were envisioned as a potential opportunity to provide early prognosis in preterm infants. However, prognosis‐based on structural brain MRI alone has shown limited success in predicting risk for neurodevelopmental problems such as motor deficits, cognitive delay, and language impairment later in life.^21^, ^22^ DTI of early white matter regions may be a more accurate biomarker, of later neurodevelopment than structural MRI which was performed in this specific case report.^18^, ^21^, ^22^, ^23^

Rose et al.^18^ concluded in their study of DTI imaging on neonatal children with very low birthweight that, it is currently not possible to predict at a near‐term age which children will experience these physical and cognitive impairments, limiting our ability to apply early interventions during critical periods of development.

## Conclusion

PNBI mortality rates are relatively low and tolerance of such injury events is remarkable. The prenatal and perinatal risk factors often do not correlate to injury types and little is known about the developmental effects on PVL white matter injuries at such a young age. In this specific case report, haemorrhagic hydrocephalus from the germinal matrix and choroid plexus region secondarily may have led to white matter PVL injury. A neurosurgical intervention was needed. Ultrasound plays little part in white matter injury detection, however, may be effective in determining long‐term survival outcomes of PNBI patients. This case report emphasises the uses of MRI in PNBI as a very useful tool for primary diagnosis and management of this complex neonatal age group and pathological process of white matter‐type injuries. Little is known about the longer‐term effects of such an injury on the development of the brain and lifelong disabilities associated with them, however studies suggest that these injuries are playing a larger role in society as advancements in modern neonatal care increase survival rates. This case report aims to increase thinking and clinical reasoning of clinicians and radiographers when presented with similar cases. I hope that this case report sparks interest and awareness of the thought processes involved in investigating PNBI.

## Conflict of Interest

The authors declare no conflict of interest.
